# Automated Assessment of Endpoint and Kinematic Features of Skilled Reaching in Rats

**DOI:** 10.3389/fnbeh.2017.00255

**Published:** 2018-01-04

**Authors:** Ioana Nica, Marjolijn Deprez, Bart Nuttin, Jean-Marie Aerts

**Affiliations:** ^1^Measure, Model & Manage Bioresponse (M3-BIORES), Department of Biosystems, KU Leuven, Leuven, Belgium; ^2^Research Group Experimental Neurosurgery and Neuroanatomy, KU Leuven, Leuven, Belgium; ^3^Department of Neurosurgery, University Hospitals Leuven, Leuven, Belgium

**Keywords:** motor cortical lesions, rehabilitation, reach-to-grasp test, image processing, kinematics

## Abstract

**Background**: Neural injury to the motor cortex may result in long-term impairments. As a model for human impairments, rodents are often used to study deficits related to reaching and grasping, using the single-pellet reach-to-grasp task. Current assessments of this test capture mostly endpoint outcome. While qualitative features have been proposed, they usually involve manual scoring.

**Objective**: To detect three phases of movement during the single-pellet reach-to-grasp test and assess completion of each phase. To automatically monitor rat forelimb trajectory so as to extract kinematics and classify phase outcome.

**Methods**: A top-view camera is used to monitor three rats during training, healthy and impaired testing, over 33 days. By monitoring the coordinates of the forelimb tip along with the position of the pellet, the algorithm divides a trial into reaching, grasping and retraction. Unfulfilling any of the phases results in one of three possible errors: miss, slip or drop. If all phases are complete, the outcome label is success. Along with endpoints, movement kinematics are assessed: variability, convex hull, mean and maximum reaching speed, length of trajectory and peak forelimb extension.

**Results**: The set of behavior endpoints was extended to include miss, slip, drop and success rate. The labeling algorithm was tested on pre- and post-lesion datasets, with overall accuracy rates of 86% and 92%, respectively. These endpoint features capture a drop in skill after motor cortical lesion as the success rate of 59.6 ± 11.8% pre-lesion decreases to 13.9 ± 8.2% post-lesion, along with a significant increase in miss rate from 7.2 ± 6.7% pre-lesion to 50.2 ± 18.7% post-lesion. Kinematics reveals individual-specific strategies of improvement during training, with a common trend of trajectory variability decreasing with success. Correlations between kinematics and endpoints reveal a more complex pattern of relationships during rehabilitation (18 significant pairs of features) than during training (nine correlated pairs).

**Conclusion**: Extended endpoint outcomes and kinematics of reaching and grasping are captured automatically with a robust computer program. Both endpoints and kinematics capture intra-animal drop in skill after a motor cortical lesion. Correlations between kinematics and endpoints change from training to rehabilitation, suggesting different mechanisms that underlie motor improvement.

## Introduction

Neural damage due to trauma, stroke, or tumor resection, for example, may induce long-term impairments that are widely studied with the use of animal models. Rodents are excellent subjects to explore the understanding of fine motor impairments that neurological damage induces in humans (Krakauer et al., 2012). In this context, the single pellet reaching task is an established method of evaluating skilled reaching in rodents, either to assess effects of motor cortex lesions (Whishaw, 2000), of models of stroke (Schaar et al., 2010; Lai et al., 2015) or to study neural mechanisms of movement in healthy subjects (Azim et al., 2014; Li et al., 2017). The test involves training rats to reach and grasp for individual food pellets. Once the animals become proficient, the test can be further used to study disruption of skilled reaching and grasping, after a cortical lesion has been induced, for example. After an initial drop of performance, with focused training of the impaired limb, animals often reach similar preoperative performance levels (Whishaw, 2000; Schaar et al., 2010). However, a prevalent question arising in clinical rehabilitation in recent years that most current studies do not address is how to study the underlying mechanism of motor improvement and whether it reflects true neural recovery or merely learning and training of new compensatory behaviors, to overcome the underlying persisting impairment (Jones and Adkins, 2015; Kwakkel et al., 2015; Hylin et al., 2017; Jones, 2017). The intact motor cortex is widely thought to be involved both in the acquisition and execution of new motor skills. However, it remains unclear how neural mechanisms related to training a new motor skill compare to recovery after neurological damage, which may involve neural repair and/or learning new compensatory behaviors to preserve that motor skill. One common way of assessing behavioral recovery is whether an endpoint has been achieved that is similar to the preoperative performance of the animal or to the performance of an intact control (Hylin et al., 2017). Such standard performance outcomes are: reaching success, first-try success, or number of attempts (Schaar et al., 2010). On the other hand, very detailed qualitative assessments have also been described in literature, where a reaching and grasping task is divided in up to ten phases, each scored independently (Whishaw, 2000; Gharbawie et al., 2005). Such detailed studies have so far revealed evidence of compensatory limb behaviors that are qualitatively different compared to true behavioral recovery. However, such approaches are yet to be used prevalently. This is due to the relative ease of evaluating the more standard endpoint features, whereas qualitative phase-based features require evaluation on a frame-by-frame basis, making the task very cumbersome. More recent kinematic studies are using multiple cameras to reconstruct 3-D trajectory of reaching (Azim et al., 2014; Guo et al., 2015), and to extend the set of biomarkers of impairment and recovery by using semi-autonomous tracking algorithms (Lai et al., 2015). However, the step to detailed automatic segmentation of movement is yet to be made. To our knowledge, no study so far aimed to compellingly study the relationship between computer-assessed kinematics and endpoint outcomes during learning and during rehabilitation.

In this study, we developed and assessed a computer program that uses image processing to monitor the kinematics of forelimb during the pellet test and infers based on it a label for the task outcome. The program can discriminate between three movement phases: reaching, grasping and retrieval and can give a label for each of them, thus discriminating between success and three types of mistakes: miss, slip, drop. We developed the algorithm on data from three rats monitored during 8 days of learning the task. We then validated it on data acquired during tests on the highly-skilled rats while they are healthy and after a motor cortical lesion in the region controlling their most dexterous forelimb has been induced. The kinematics revealed an individual strategy to optimally accomplish the task during the training phase. Moreover, the cortical lesion altered the fine spatio-temporal structure of reaching, grasping and retraction phases, triggering compensatory behavior, which cannot be captured just by monitoring the percentage of successful attempts, but also the type and distribution of errors. Thus, the analysis revealed the need for longitudinal, intra-animal studies that focus on individualized kinematics of movement, where improvements in endpoint measures are accompanied by a significant reduction in subjects’ trajectory variability. Correlation analysis between endpoint features and kinematics revealed different patterns of linear relationships between the training and the post-lesion rehabilitation stages, underlying strategies of performing the reach and grasp task.

Thus, this study emphasizes the need for individualized methods of monitoring performance that fuse traditional endpoint features with kinematics of movement and raises questions on how such variables help explain improvement and change our definition of recovery, be it due to true neural repair or learned compensatory behavior.

## Materials and Methods

### Subjects

Three male Sprague-Dawley rats weighing ~300 g were housed individually in standard plastic cages (light on a 14:10 h cycle beginning at 7:00 AM; room temperature 22°C). The animals were 8 weeks old at the beginning of the experiment. Five days prior to the start of training, the animals were gradually food deprived to reach 90% of their body weight by the start of the experiment. The animals were fed standard laboratory chow (1 g per 50 g of body weight) after the testing period each day. By the time testing began at 10:00 AM, all three rats had no remaining food in their cage, thus being sufficiently restricted and motivated to perform the task. The experiments were approved by the Animal Ethics Committee KU Leuven, and all procedures were in accordance with the Belgian and European laws and guidelines for animal experimentation, housing and care (Belgian Royal Decree of 29 May 2013 and European Directive 2010/63/EU on the protection of animals used for scientific purposes of 20 October 2010, project number: P218/2014).

### Reaching Task

**Figure 1:**
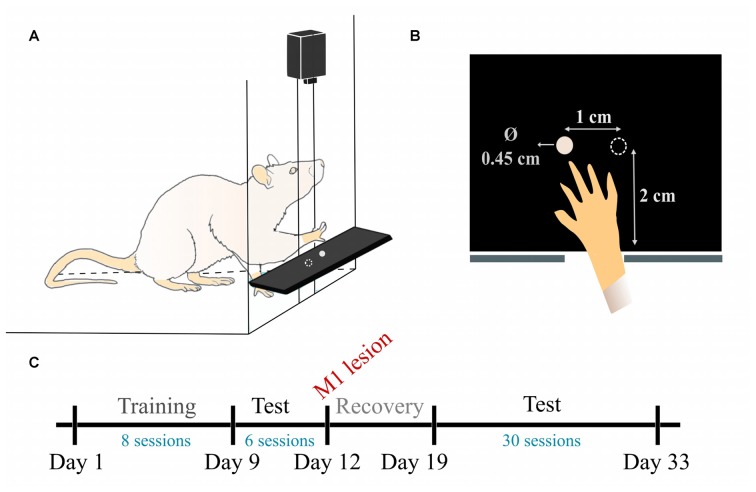
Pellet test setup and experimental timeline. **(A)** Schematic view of the reaching box. **(B)** Top-view illustration of the pellet placement with respect to the slit. **(C)** Timeline of the data experiment and data acquisition. During training (days 1–8) one test/day was recorded. During pre-lesion (days 9–11) and post-lesion (days 19–33) testing periods, two tests/day were recorded.

The pellet test setup and training paradigm were based on established methods (Whishaw, 2000). The animals were trained to reach and grasp in a clear Plexiglas reaching box (19.5 cm long, 8 cm wide, and 20 cm high), with a 1 cm-wide slit in the anterior side. A plastic shelf (8 cm long, 6 cm wide, 2 cm tall) was mounted in front of the box. Two indentations were created in the shelf, 2 cm away from the slit, and symmetrical to its edges, spaced 1 cm away from each other (Figure 1A).

Two days before start of training, the rats were habituated to the cage. Pellets were initially available on the cage floor and within tongue distance on the shelf. Pellets were gradually placed farther away on the shelf until the rats were forced to reach to retrieve the food. The pellets were placed in both indentations initially, allowing the animals to display forelimb preference.

Since day 1, once we started recording the training phase, we solely used the indentation contralateral to the preferred forepaw, which allows the rat to obtain the pellet with the most dexterous forelimb and not with the tongue or the other forelimb (Figure 1B). The training consisted of reaching for 20 food pellets (Dustless precision pellets, 45 mg, Bio-Serv, Flemington, NJ, USA) until they were all consumed. The experimenter re-placed the pellet in the indentation as many times as needed before the rat managed to successfully retrieve it. The training session was performed once a day, for 8 days.

Once the rats achieved a mean success rate higher than 40% over three consecutive days, we proceeded to test them. A test session differed from a training session in that each of the 20 pellets was offered only once. The rat was allowed to reach for the pellet until it either displaced it, in which case the experimenter took it away, or until it could retrieve it through the opening, into the box. If the rats missed the target or touched the pellet without knocking it away from the indentation, they were allowed to continue. We collected six tests for each animal, during 4 days (days 9–12). The average success rate was 59.7% (±5.7%), comparable to other similar behavior studies (Gharbawie et al., 2005; Alaverdashvili and Whishaw, 2008; Alaverdashvili et al., 2008). Thus the animals were considered trained for the task and we proceeded to inducing cortical lesions.

### Surgery

A lesion in the area of the primary motor cortex was induced on day 12. Rats were anesthetized with a mixture of ketamine (Nimatek^®^) and medetomidine hydrochloride (Narcostart^®^). A craniotomy over the forelimb area of the primary motor cortex contralateral to the preferred forelimb (right hemisphere in one, left hemisphere in two animals) was made, using coordinates: 1.5 mm posterior to 5 mm anterior to Bregma, and 0.5 mm to 4.5 mm lateral to Bregma, after which the exposed brain tissue was aspirated to a depth of 1.5 mm. Lesions were made to include the rostral and caudal forelimb areas. We defined these coordinates in a preliminary mapping experiment performed in animals of the same sex, age and weight that had been trained for a similar skilled task (data not published).The animals were returned to their home cages, and the pellet test was resumed on day 19, following 1 week of rest.

### Histology

Rats received a pentobarbital overdose (Nembutal, CEVA Santé Animale, Belgium; 3 ml i.p.) after which they were perfused intracardially with a solution of 10% sucrose (D(+)-Saccharose, VWR International BVBA, Belgium), followed by a 4% formaldehyde solution (37% dissolved in water, stabilized with 5%–15% methanol, Acros organics, Belgium; 10× diluted in DI water). The brain was removed, embedded in paraffin and was then sliced with a microtome (Leica Biosystems GmbH, Germany) to obtain 10 μm slices. Slices were stained with cresyl violet (0.5% cresyl violet acetate in dH_2_O, Merck KGaA, Germany), and were then microscopically inspected and visually compared to a Paxinos stereotactic atlas (Paxinos and Watson, 2005) to determine the lesion extent and location (with the observer blinded for group allocation). Lesion depth and width were estimated based on the deepest and widest point of the lesion, respectively, and results are summarized in Table 1. On average, the lesions extended between 1.12 ± 0.99 mm posterior to 4.28 ± 1.6 mm anterior and 1.2 ± 0.35 mm medial to 2.6 ± 0.2 mm lateral. The lesions were rather large, but representative of those described previously (Whishaw, 2000).

**Table 1:** Histology results.

	Lesion location (mm)						Lesion extent (mm)		
Rat	A	P	M	L	D	V	A-P	M-L	D-V
R1	2.52	1.92	1	2.4	0.2	3	4.44	1.4	2.8
R2	5.64	0	1	2.6	0.4	3	5.64	1.6	2.6
R3	4.68	1.44	1.6	2.8	0.6	2.6	6.12	1.2	2
Mean	4.28	1.12	1.2	2.6	0.4	2.87	5.4	1.4	2.47
SD	1.60	0.99	0.35	0.20	0.2	0.23	0.87	0.2	0.42

*Lesion location and lesion extent are determined based on the most anterior point (A), most posterior point (P), medial (M) and lateral (L) borders at the widest point and dorsal (D) and ventral (V) borders at the deepest point. Lesion extent is shown in terms of length (antero-posterior spread, A-P), width (medial-lateral spread, M-D) and depth (dorsal-ventral spread, D-V). All values represent distances (mm) to Bregma*.

### Video Recording and Timeline of Data Acquisition

We performed top-view recordings of the task with a Sony DSRPD100 camera (30 Hz sample rate, 120° wide angle, resolution 1920 × 1080) placed ~10 cm above the reaching table. As shown in Figure 1C, data was collected in three phases: training, pre-lesion testing and post-lesion testing. In total, we collected eight training phase sessions, as the rats learned to execute the task (days 1–8), 6 tests pre-lesion, when the animals were healthy and well skilled for the task (days 9–12) and 30 sessions in post-lesion rehabilitation, as the rats gradually improved their skill over 15 days (days 19–33).

### Pre-processing

To remove the effect of lens distortion, we determined camera distortion parameters with a checkerboard calibration pattern, by using Matlab Single Camera Calibration App (Computer Vision System Toolbox, Mathworks, Natwick, MA, USA). These parameters were further used in the video monitoring routines, to undistort each frame before analysis. To account for millimeter variations in the way the camera was positioned for each test and to reconstruct 2-D world coordinates of the forelimb, we determined the pixel/centimeter ratio of each video, by taking as reference the reaching table, a black object of exactly 5 cm width.

### Behavioral Monitoring

We hypothesized that both training and the motor lesion would not only impact the success rate, which is quantifiable manually, but it would also alter the fine spatio-temporal structure of the reaching, grasping and retraction phases. The objective of the behavioral monitoring was thus two-fold: to assess movement kinematics but also to use kinematics to infer the outcome of the test, be it success or error. Furthermore, we hypothesized the type of error would provide insight into the mechanisms of learning or rehabilitation, so we extended the possible classes of errors to three categories: miss, slip, drop. All video analyses were performed in Matlab, using the Computer vision toolbox (in pre-processing and step (i)) and Image processing toolbox (in step (i)), along with custom designed functions.

**Figure 2:**
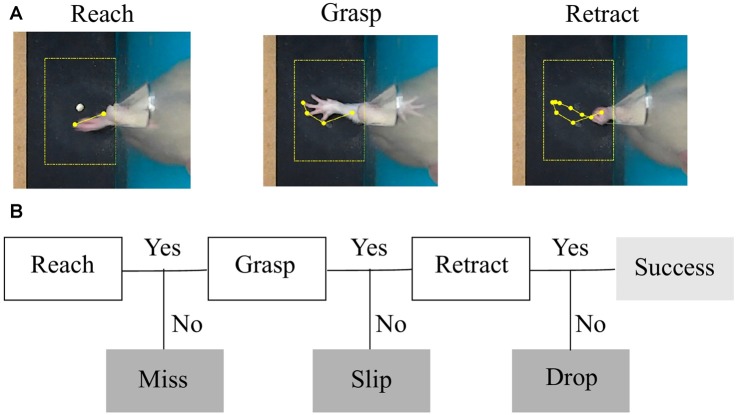
Task phases and error categories. **(A)** The three phases of a successful trial captured by a top-view camera. The region of interest centered around the detected pellet and the trajectory of the forelimb tip are plotted in yellow. **(B)** Decision tree that treats each phase as a condition for success and labels the type of outcome.

Behavioral monitoring was performed off-line, in three steps. In step (i), the algorithm analyzed all frames in a recording session to identify and digitize location of the pellet and the forelimb tip along with features of the forelimb’s shape. Rats identify food by olfaction (Alaverdashvili and Whishaw, 2008). However, we observed instances when rats reach towards an empty indentation, either before the pellet was placed in the indentation by the experimenter, or after they had displaced it. Consequently, we programed the algorithm to first identify the pellet with respect to the indentation, so as to ensure that such attempts were excluded and only reaches aimed at a correctly positioned pellet were quantified. The position of the indentation was defined manually for each video, since the contrast was not high enough to determine it by means of image processing. This was the only manually required input. A rectangle of size 0.5 × 0.5 cm centered on the indentation is generated as the preliminary region of interest (ROI). In this preliminary ROI, the pellet can be identified by means of pixel intensity discrepancy, since the pellet is almost white in color, against the dark background of the reaching table (Figure 2A). However, the metallic forceps used to place the pellet in the indentation was also occasionally transiting this region, so to ensure such noise is excluded, an extra condition was imposed. Once an object was detected, its perimeter, *P* and area *A* are used to test if the object is round, using circularity *C* defined as

$$C\text{ = }P^{\text{2}}\text{/(4}\pi A\text{)}$$

*C* has a value of 1 for circles and more than 1 for all other objects. Roundness and not area was the only condition for detecting the pellet, since bigger objects would not fit in the preliminary ROI.

Once the pellet was confirmed to be in the indentation, the search for the forelimb was triggered, in an ROI of 4 × 6 cm centered on the detected pellet. The size of the ROI was devised to monitor reaching once the forelimb passed through the slit. The activity of the forelimb in the cage was obscured by the animal’s head and it could not be reliably quantified. The cropped image was converted to a binary image. Because the limb of the rat is light in color, it can easily be identified against the black reaching table without the need to use skin markers (Figure 2A). Using the Matlab function “regionprops” (Image Processing Toolbox), forelimb shape features like centroid, area, orientation, minor and major axes, x- and y-coordinates of the tip were assessed. Timestamps for the position of the pellet and forelimb tip, the coordinates of the indentation and the ROIs, and forelimb shape features were saved.

**Figure 3:**
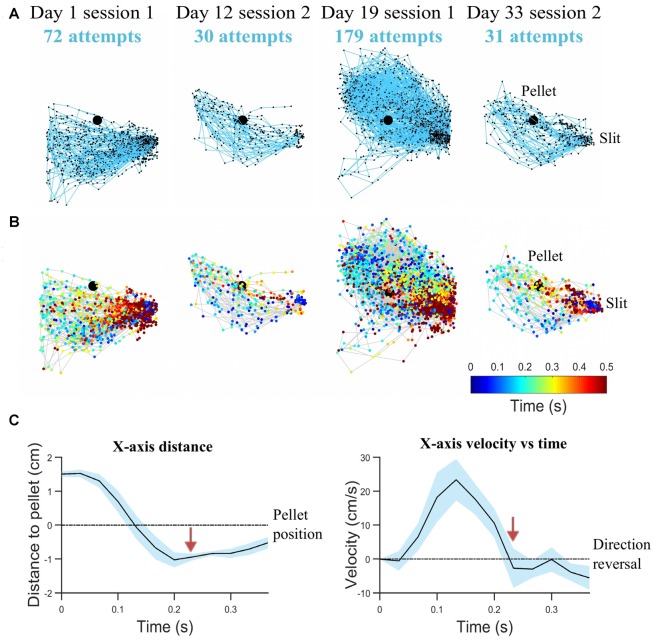
Reaching kinematics. **(A)** Tracked forelimb trajectories capture skill variability. Trajectories of all reaching, grasping and retraction attempts are plotted for a representative rat on start of training (day 1), last test before lesion induction (day 12), first rehabilitation test (day 19), last rehabilitation test (day 33). All trajectory coordinates are normalized with respect to the pellet (represented as the black filled circle). The slit, from which movement is initiated and completed, is indicated on the right-hand side. **(B)** Corresponding spatio-temporal representation for trajectories represented in **(A)**. Each trajectory coordinate is plotted as a colored circle, where the color represents the time from the start of the attempt (see Legend). **(C)** Mean kinematics from a representative rat captured between the start of a reaching attempt and grasping (average over 20 attempts). Shaded regions represent 95% confidence intervals. Transition from reach to hovering phase delineated by red arrows, as velocity decreases. Velocity crossing of zero indicates direction reversal. For clarity, variations of velocity and distance with time are shown with respect to x-axis direction.

In step (ii), pellet and forelimb coordinates are analyzed as time series, to determine the interval of a reaching attempt. Figures 3A,B shows examples of reaching trajectories reconstructed based on forelimb tip coordinates between the timestamps for start of reach and grasping. These timestamps were extracted in step (ii). The most robust feature to detect an attempt was the maxima in the time series of forepaw shape area, marking the moments of maximum forelimb extension. The start of an attempt was defined as the first frame before the forepaw area started increasing monotonically. This condition was imposed rather than just detecting the timestamp when the forelimb enters through the box slit and is detectable in the ROI, so as to exclude intervals when the rat was resting its forelimb on the reaching table or was hesitating before moment of attack. The moment of grasping was detected in the frames following the maximum forelimb extension, based on velocity profile (Figure 3C). A decrease in speed succeeded the change in x-axis direction, as the rat retrieved the forepaw from maximum extension position and hovered above the pellet location before touching it. While reaching towards the pellet is quite a uniform and repeatable phase of the attempt, the behavior following grasping was variable. Thus, in this stage only the timestamps for start of reach and grasping (hovering) were detected. Kinematic features based on the reaching phase were extracted in this step, such as maximum speed of reaching, mean speed of reaching, total reaching length, mean peak extension, variability of reaching trajectories and convex hull (see “Behavioral Endpoints” section).

**Figure 4:**
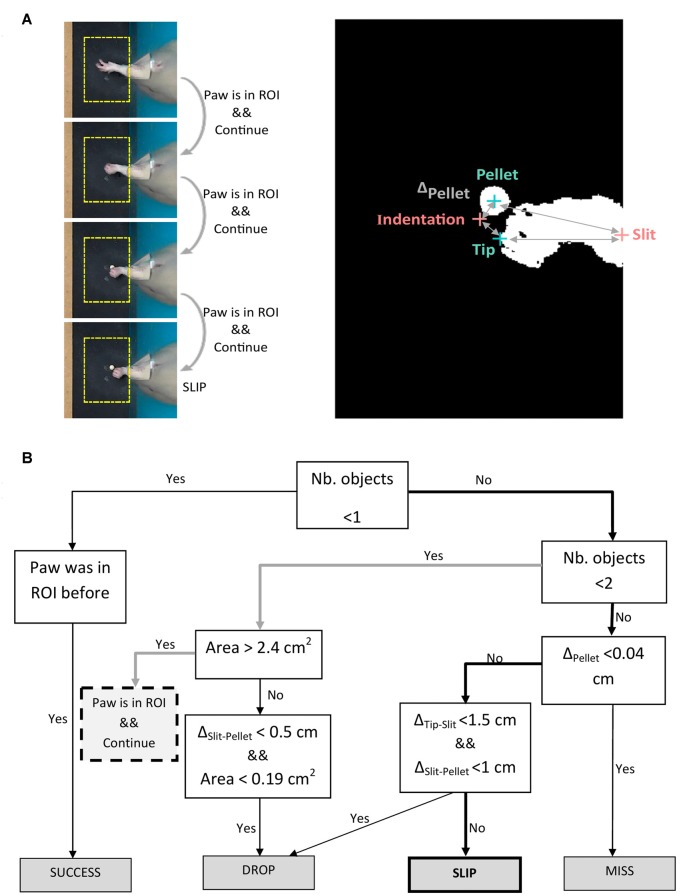
Algorithm for outcome labeling. **(A)** Left panel: example of algorithm decision on four frames succeeding maximum forepaw extension. Right panel: binary image of the frame in which the slip occurs, showing detected objects (forepaw tip, pellet) and fixed landmarks (indentation, slit). **(B)** Decision tree algorithm iterated on the frames succeeding maximum forepaw extension. Description of abbreviations: Δ_Pellet_ = distance between indentation and current pellet position; Δ_Tip-Slit_ = distance between forepaw tip and slit; Δ_Slit-Pellet_ = distance between pellet and slit.

In step (iii), a decision tree was implemented to assess the outcomes of each attempt. As explained in step (ii), we aimed to segment movement in three phases: reaching, grasping and retraction. Forelimb position and direction of movement with respect to the pellet was used by the algorithm to label the outcome of each attempt into four possible categories: miss, no grasp, drop and success (see Figure 2B). We used the timestamps for start to reach and hovering/grasping detected in step (ii), to create a decision tree algorithm that labels attempt outcome after the rat reached for the pellet (Figure 4B). Starting from the first frame where hovering is detected, the algorithm checks how many objects are detected in the ROI for the next 25 frames (~0.8 s) or until no objects is detected, meaning the forelimb was completely retracted. Assuming the rat missed the target, the position of the pellet will be unchanged as the forelimb retracts. We excluded a miss when Δ_Pellet_, the distance between indentation and current pellet position, didn’t exceed a value of 0.04 cm. If the rat touches the pellet without grasping it, or grasps it correctly, but drops it while retracting, the pellet will again become visible in the ROI, in a changed position. The algorithm detects a drop if the position of the pellet is within 1 cm away from the slit (Δ_Slit-Pellet_ < 1). The other cases are taken as a slip. An example of how the algorithm detects a slip is pictured in Figure 4A. The algorithm gives a label of success if the pellet is at no time detected in the ROI until the forepaw is completely retracted out of the ROI.

### Behavioral Endpoints

All endpoint and kinematic parameters were calculated individually for each animal and for each session.

#### Task Outcome

As shown in Figure 2A, the reaching phase consisted of an extension of the limb within the cage slit and beyond the position of the pellet. The limb then returned and hovered above the pellet with a decreased speed. Then, the grasping phase occurred. In the retraction phase, the animal returned the forelimb holding the pellet back in the cage. These were the movements captured with our setup. We did not capture behavior inside the cage, e.g., the start of reaching or the eating of the pellet. However, the phases described were still informative as to the skill of reaching and grasping.

As described in “Behavioral Monitoring” section, the instantaneous position of the forelimb, its velocity profile (speed and direction of the tip of the forelimb) along with the position of the pellet with respect to the indentation allowed us to develop a decision tree algorithm that identified phases of movement and labeled the outcome of the attempt (Figure 2B). Based on total number of attempts executed in the session, we calculated miss, slip, drop and success percentages as endpoint measures of one session.

#### Variability

Dynamic time warping is a distance measure that allows for time shifting and can thus match similar shapes even when they have a time phase difference. Since reaching trajectories were of varying time lengths, we calculated intra-animal variability by using pairwise dynamic time warping distance between all trajectories recorded in one session.

#### Convex Hull

The convex hull of one training session is the area containing all x-y coordinates tracked during that session. This surface was drawn between the most extreme points of the forelimb coordinates. While variability is a function of time-aligned space coordinates, the convex hull provides solely space information, as it quantifies the spread of the reaching trajectories performed during one session.

#### Mean and Maximum Speed

Mean speed was averaged during each individual reach, then averaged between all reaches in one session. Maximum speed was taken as the absolute maximum speed achieved between all reaches in one session.

#### Reaching Length

The length of each reaching trajectory, calculated between the start of the reach and the positioning of the paw above the pellet was taken as the reaching length. All reaching length values were averaged over one session.

#### Peak Limb Extension

As shown in Figures 3A,B, the animals extended their limb beyond the position of the pellet, before returning to grasp it. Peak limb extension is the length of the forelimb at the moment of maximum limb extension. All values were averaged over attempts within one session. While the pasta matrix test directly assesses physical limits of reaching, with the reach-to-grasp test, the pellet is well within the limits of reaching for each rat. Thus, shorter peak limb extensions should not be interpreted as a proof of impairment, since the animal might have a more directed reaching strategy even as an intact subject. However, changes in peak limb extension might signal changes in strategy of reaching, which in turn may reflect compensatory behavior.

### Statistical Analysis

Statistical analyses were performed using MATLAB with a significance level α = 0.05. To assess the effect of the lesion on performance and the effect of the rehabilitation, we created two groups with the post-lesion data, comprising, respectively, the first six tests after lesion induction (days 19–21) and the last six tests of the rehabilitation (days 31–33). The third set included the six tests recorded pre-lesion (days 9–12) when the rats were well skilled. We compared outcome percentage and kinematic parameters in these three sets using the non-parametric Kruskal-Wallis test with Dunn-Sidak *post hoc* pairwise comparisons. To test linear relationships between kinematic parameters and task outcome, we calculate Pearson coefficient. *P*-values were adjusted with a Bonferroni correction.

## Results

### Task Outcome

#### Labeling Accuracy

**Figure 5:**
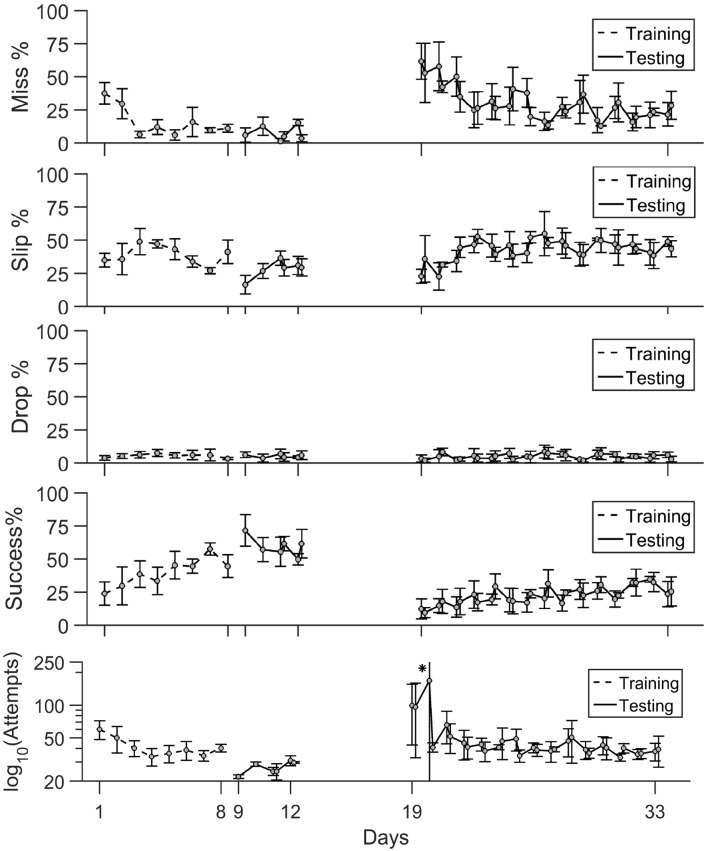
Timeline of task phase outcome. The percentage of misses, slips and drops together with success rate and number of attempts are shown during training (days 1–8), testing (days 9–11) and rehabilitation (days 19–33). Mean values ± standard deviation are shown. (*) In panel (v), the number of attempts is represented on a log scale. The mean value ± standard deviation on day 20 were [8.5 329.5].

We assessed accuracy of the labeling algorithm by manually scoring a subset of the videos for each experimental stage: the three acclimation sessions from day 0, before training began, were used together with 38% of videos from training phase (days 1–8) to develop and validate the training set. We included the three acclimation sessions in order to capture more extreme behavior and make the algorithm more robust. To test the algorithm, 33% of videos from pre-lesion tests and 20% of videos of post-lesion testing were used. The percentages are equal for all animals. This allowed us to estimate the rates of predicted labels with respect to actual labels, and the results are presented in confusion matrices (see Tables 2–4). The reason for assessing each dataset separately is that the distribution of labels differs from one stage to the next. Figure 3A captures the kinematics of impairment after lesion. As seen in Figure 5, the animals are very successful in the pre-lesion stage, while exhibiting high percentages of misses or slips in the post-lesion testing. Moreover, since the rats exhibit task-unrelated behavior especially in the beginning of the training period and at the start of the rehabilitation period respectively, we defined an additional label, “Other” for all non-attempt movements captured by the forelimb monitoring algorithm that could be discarded by the labeling routine. These include movements of the forelimb in the region of interest that are not related to or not according to the instructions for the skilled reaching task, like keeping the forelimb stationary on the table, or grasping the pellet straight from the forceps before the experimenter could position it on the indentation. The overall accuracy for development and validation sets was 92% (Table 2). Similar accuracy was obtained for the pre-lesion test (86%, Table 3) and the post-lesion test (92%, Table 4).

**Table 2:** Confusion matrix with correctly and incorrectly classified attempts from the training phase (days 1–8).

**True class**	Miss	**86**	3	0	1	0	96%
	Slip	2	**182**	0	8	0	95%
	Drop	0	2	**18**	5	0	72%
	Success	2	2	4	**163**	2	94%
	Other	1	3	0	4	**20**	71%
							= TPR
		99%	97%	99%	94%	99%	= Specificity
		Miss	Slip	Drop	Success	Other
		**Predicted class**				

*The algorithm outcome was validated on 38% of videos selected randomly from the training phase. Five labels (N = 509) are correctly classified in 92% of cases. TPR = true positive rate = sensitivity. In bold are the correctly classified instances (true positives)*.

**Table 3:** Confusion matrix with correctly and incorrectly classified attempts from the test phase (days 9–11).

**True class**	Miss	**11**	2	0	0	0	85%
	Slip	1	**49**	0	6	0	88%
	Drop	0	0	**10**	1	0	91%
	Success	0	1	0	**71**	2	96%
	Other	0	4	0	7	**2**	15%
							= TPR
		99%	93%	100%	85%	98%	= Specificity
		Miss	Slip	Drop	Success	Other	
		**Predicted class**				

*The algorithm outcome was validated on 33% of videos selected randomly from the test dataset. Five labels (N = 167) are correctly classified in 86% of cases. TPR = true positive rate = sensitivity. In bold are the correctly classified instances (true positives)*.

**Table 4:** Confusion matrix with correctly and incorrectly classified attempts from the rehabilitation phase (days 19–33).

**True class**	Miss	**194**	4	1	1	1	97%
	Slip	9	**295**	1	8	1	93%
	Drop	0	0	**31**	0	0	100%
	Success	0	6	4	**117**	6	88%
	Other	0	4	0	9	**50**	79%
							= TPR
		98%	97%	99%	97%	99%	= Specificity
		Miss	Slip	Drop	Success	Other	
		**Predicted class**				

*The algorithm outcome was validated on 20% of videos selected randomly from the rehabilitation phase. Five labels (N = 747) are correctly classified in 92% of cases. TPR = true positive rate = sensitivity. In bold are the correctly classified instances (true positives)*.

#### Training Phase

Early training sessions exhibited a high number of attempts and high percentages of misses (Figure 5). The rats became more successful in locating the pellet and the rate of misses decreased at under 10% in only 3 days of training. The precision of grasping stayed variable between days as slips remain the main type of error the animals made throughout the training period. The percentage of drops was constantly under 5%. Success rate steadily increased with each day of training, and it became the main test outcome in the last 3 days of training at rates of 45%–50%.

#### Pre- vs. Post-lesion Tests

**Figure 6:**
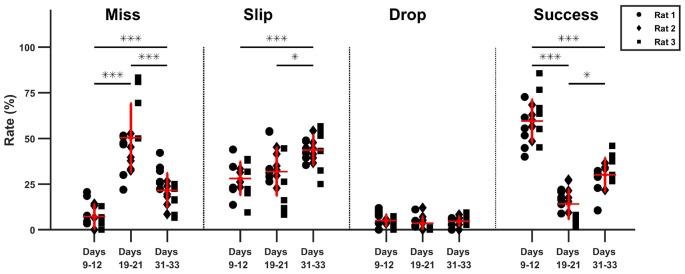
Comparison of task phase outcome. The percentage outcome is compared between pre-lesion testing, the start and the end of rehabilitation (*p < 0.05, ***p < 0.01).

As seen in the timeline of outcomes from Figure 5, a drop in performance after lesion induction occurred, but rats progressively became more successful during the 15 days of post-lesion testing. We compared the rate of misses, slips, drops and success between three phases: days 9–11 when the healthy animals were well skilled for the task, day 19–21, the first on resuming training when the effect of the lesion was most severe, and days 31–33, to test the effect of rehabilitation. The results of the comparisons along with individual data points are summarized in Figure 6.

The relative rate of missed attempts increased significantly from 7.2 ± 6.7% pre-lesion to 50.2 ± 18.7% post lesion (*p* ≪ 0.01) and remained significantly high at 21.6 ± 9.3% with respect to pre-lesion testing after 15 days of rehabilitation (*p* < 0.01), although it also decreased significantly with respect to the beginning of rehabilitation (*p* < 0.01). The relative rate of slips did not change significantly after lesion induction: 28.1 ± 13.1% pre-lesion and 31.9 ± 8.9% post-lesion (*p* > 0.05). However, the rate of slips increased by the end of rehabilitation to 43.63 ± 8.1%, significantly higher with respect to pre-lesion tests (*p* ≪ 0.01), and to the start of rehabilitation (*p* = 0.02). There was no significant change in drop percentage, which remained under 5 ± 3.2% during all three testing phases. The rate of success decreased significantly from a mean of 59.6 ± 11.8% pre-lesion to 13.9 ± 8.2% post-lesion (*p* ≪ 0.01). Success rate increased to 30 ± 9.2% at the end of rehabilitation, significantly higher with respect to beginning of rehabilitation (*p* = 0.011) but still significantly below the levels before lesion induction (*p* ≪ 0.01). For all outcome labels, no between-animal differences were significant.

### Kinematics

As with outcome labels, kinematic features were compared among three phases of the experiment using Kruskal-Wallis test with Dunn-Sidak *post hoc* for pairwise comparisons. Based on observed reaching trajectories, it seems that rats developed individual strategies for reaching, so additionally, we tested in the same way if kinematics can help discriminate between the animals.

#### Variability

We tested intra-rat variability, finding a significant increase in the beginning of rehabilitation at 11.5% (±3%) from pre-lesion tests, where the mean was 9.2% (±1.6%). By the end of rehabilitation, the mean was 9.2% (±1.1%), similar to that of the pre-lesion tests. Rat 1 exhibited values significantly lower than rats 2 and 3 (*p* ≪ 0.01).

#### Convex Hull

The convex hull increased significantly (*p* ≪ 0.01) from 4.2 (±0.6) cm^2^ pre-lesion to 6.4 (±1.8) cm^2^ post-lesion, but the difference disappeared by the end of rehabilitation, when the mean convex hull was 4.3 (±0.3) cm^2^. There were also differences between rats, with rat 1 trajectories (4.1, ±0.8 cm^2^) having significantly (*p* = 0.03) lower convex hulls than rats 2 (5.1 ± 1.1 cm^2^) and 3 (5.6 ± 2 cm^2^). There were no differences between rats 2 and 3.

#### Mean Speed and Maximum Speed of Reaching

Mean speed of reaching decreased at the end of rehabilitation with respect to the start of rehabilitation, but the difference was not significant. There were no significant differences with respect to pre-lesion tests. There were also no significant differences between rats.

Maximum speed decreased significantly (*p* = 0.05) from 45.8 (±3.5) cm/s pre-lesion to 43.4 (±4.2) cm/s in the beginning of rehabilitation, but the difference was not significant by the end of rehabilitation. Rat 2 had a significantly faster maximum speed (*p* < 0.01) at 46.7 (±3.9) cm/s than rat 3 (42.3 ± 2.2 cm/s), but not rat 1 (43.2 ± 3.5).

#### Length of Trajectory

Although there were no significant differences in trajectory length (4.17 ± 0.02 cm) between the phases of the experiment, there were significant differences between rats (*p* < 0.01), as rat 2 shows significantly shorter trajectories (3.9 ± 0.3 cm), than rat 1 (4.4 ± 0.3 cm, *p* < 0.01) and rat 3 (4.2 ± 0.3 cm, *p* = 0.03).

#### Peak Limb Extension

Peak limb extension decreased significantly (*p* < 0.01) in the beginning of rehabilitation (2.8 ± 0.2 cm) with respect to pre-lesion (3.07 ± 0.23 cm) and remained significantly lower (*p* = 0.01) at the end of rehabilitation (2.8 ± 0.18 cm). There were no differences in peak limb extension between animals.

### Task Outcome and Kinematics during Training and Rehabilitation

Performance improved in both the training phase and the post-lesion phase, with similar trends, as shown in Figure 5: success rate increased as miss percentage lowers, slip is consistently the most prevalent type of mistake, while drop rates are insignificant. To examine if kinematics help explain improvement in task outcome and if the mechanisms for improvement are similar in training and in rehabilitation, we calculated correlations between kinematic features and behavioral endpoints independently for each phase. We took the Pearson coefficient as a measure of correlation and we set a significance level of *p* = 0.05. All correlation strengths for non-significant pairs of features were subsequently set to zero.

**Figure 7:**
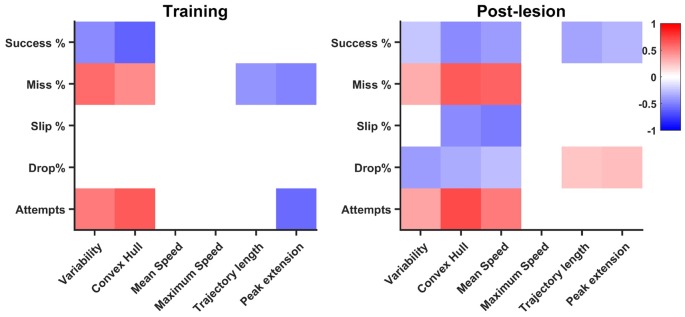
Correlation of endpoint outcome with kinematics. Significant (*p* < 0.05) correlation pairs are represented. The pattern of correlations increases in the post-lesion dataset (days 19–33) with respect to the training phase (days 1–8).

The training phase revealed a number of nine significantly correlated pairs of features, with an increase in the rehabilitation stage to 18 pairs, suggesting overall a different contribution of kinematic features to task success (see Figure 7). Number of attempts was positively correlated with variability (*R* = 0.51) and convex hull (*R* = 0.65) in the training phase, a relationship that is maintained during rehabilitation (*R* = 0.37 and *R* = 0.72, respectively). The negative correlation with peak limb extension (*R* = −0.58) during the training phase became insignificant during rehabilitation, while additionally, a positive correlation with mean speed (*R* = 0.53) occurred during rehabilitation. There was a similar trend with miss percentage, where positive correlations with variability (*R* = 0.57) and convex hull (*R* = 0.46) in the training phase were maintained during post-lesion phase (*R* = 0.33 and *R* = 0.64, respectively) during post-lesion phase. Additionally, miss rate was positively correlated with mean speed during rehabilitation (*R* = 0.62). Interestingly, as trajectory length and peak limb extension increase during training, miss percentage decreases (*R* = −0.41 and *R* = −0.49, respectively). These negative correlations were not maintained during post-lesion tests, suggesting that precision of reaching was achieved in different ways in the two phases. While slip rate was not correlated with any kinematic features during training, it was negatively correlated with convex hull (*R* = −0.45) and mean speed (*R* = −0.51) during post-lesion tests. Drop rates were not significantly correlated with any kinematic features during the training phase, but were negatively correlated with variability (*R* = −0.38) and convex hull (*R* = −0.32) in the rehabilitation stage and weakly correlated with trajectory length and peak limb extension (*R* < 0.3). Success rate was negatively correlated with variability (*R* = −0.44) and convex hull (*R* = −0.61) in the training phase, which weakened during rehabilitation (*R* = −0.23 and *R* = −0.46, respectively) and additionally, small negative correlations with mean speed (*R* = −0.38), trajectory length (*R* = −0.37) and peak limb extension (*R* = −0.3) arose during this phase.

## Discussion

The present study demonstrates the feasibility of automatically tracking reaching and grasping without additional need of skin markers. Monitoring the forelimb from top-view recordings generated a kinematic profile of movement. Based on it, we developed necessary conditions for the algorithm to segment the task into reaching, grasping and retraction. Top-view monitoring proved sufficient to reliably detect the outcome of the three movement phases and thus distinguish between the following types of errors: miss, slip and drop along with overall task success. The Matlab algorithms were developed on a dataset consisting of training sessions and the overall accuracy achieved on new datasets was of 86% (pre-lesion test set) and 92% (post-lesion test set).

Combinations of front-view and lateral-view cameras (usually infra-red or near-infra-red sensitive), have been used in recent studies to reconstruct 3-D kinematics of reaching and grasping in rodents. While kinematic assessment could be achieved autonomously (using infra-red reflective markers to track the forelimb, in Azim et al., 2014) or semi-autonomously (tracking with machine learning techniques, while segmentation of movement is achieved manually by Guo et al., 2015), these studies didn’t extend their automatic tracking to more descriptive measures of movement and no measure of algorithm performance is reported. Lai et al. (2015) used a side-view camera together with a tilted mirror to reconstruct reaching trajectories in a sagittal and coronal plane and extended types of kinematic parameters to characterize reaching, grasping and retrieval, but did not use kinematics to further extend or explain endpoint measures. Esposito et al. (2014) proposed a segmentation of movement and types of errors similar to the one we used, but the forelimb tracking and classification are achieved manually. Two recent studies (Wong et al., 2015; Ellens et al., 2016) developed a fully automated apparatus to perform the pellet test and assess success or failure online.

On the other hand, very detailed, but manually scored qualitative measures have been proposed, either to segment reaching and grasping movement into 10 phases (Whishaw, 2000, 2002; Whishaw et al., 2004), to study individual digit movement (Alaverdashvili and Whishaw, 2008) or types of gestures that arise with impairment (Alaverdashvili et al., 2008). These qualitative approaches are very informative and capture adaptation strategies to induced impairments, but given the burden of manual scoring, they have not been widely adopted. The main findings of these studies point towards decreased wrist rotation, additional adaptive body rotation, decreased individual movement of digits and increase in gestures with impairment, highlighting the role of compensation after neural damage.

In comparison, in our study we developed an algorithm that uses image processing to semi-autonomously reconstruct the kinematics of reaching, which are then further used to gain insight into the quality of movement, by segmenting attempts into three phases of movement. We used one camera placed on top to capture x-y-coordinates of movement (transverse plane). A front view analysis is very difficult to implement with a computer routine, given the lack of contrast between the paw of the rat and its body, both light in color. A side view recording, while offering a better glimpse at the activity of the limb inside the cage, and in the sagittal plane, would miss movement in the transversal plane, which proved very informative in our study. In comparison with most studies cited, we used a rather low sampling rate of 30 Hz. This caused occasional blurring, especially during the reaching phase, when the speed of the forepaw was maximum. However, since we quantified trajectories based on the tip of the paw instead of the centroid, the blurring did not affect the result during the reaching phase. We did not observe blurring during retraction in the frames we manually quantified for scoring and validating the result of the classification, possibly because the speed of the paw decreases during retraction (Figure 3C). Higher sampling rates would also provide more accurate estimates of kinematic features, especially when estimating maximum speed. When the animals move fast, we might be underestimating the distance between two consecutive forelimb coordinates, due to the low frame rate. Since top-view recordings with a rather low sampling rate were sufficient in our study to reach an overall accuracy percentage of 86%–92% in scoring outcome, it would be informative to assess what further information can be extracted from an additional dimension at a higher time resolution, possibly features like rotation of the wrist, which can lead to further movement segmentation and outcome classification.

While using the classical single pellet reaching test setup (Whishaw, 2000), we did not force the rat to execute true grasping rather than dragging the pellet and we also did not distinguish between dragging and grasping with our algorithm. We attempted to quantify grasping by assessing the minor-axis length of the forepaw shape (the width), but this feature didn’t prove robust enough (data not shown). However, this strategy may be used robustly if bigger variations in shape width can be captured, for example while testing non-human primates or humans performing similar reaching and grasping tests. Also, while animals exhibited dragging rather than grasping since the training phase, we did not associate dragging with impairment. While not eliminating dragging, detecting it would be possible by increasing the frame rate, which may provide more robust results. In other studies that automatically quantify kinematics of the reach and grasp test, dragging was detected from sagittal plane recordings (Lai et al., 2015). One possibility to eliminate dragging would be a change of experimental setup and such a solution has been proposed by Hays et al. (2013), who developed a novel setup, called the isometric pull task, to quantify both reach-to-grasp dexterity and forelimb strength (Sloan et al., 2015).

Analysis of phase outcome captured disruption in motor skill and prevalence of abnormal behavior, as rates of misses increased seven times between pre- and post-lesion tests. By the end of rehabilitation, the percentage of misses had decreased, but it was still three times higher than in pre-lesion tests. Significant increases of slip percentage during rehabilitation were also observed. A study focused on gesture analysis (Alaverdashvili et al., 2008) reported an increased number of gestures with rehabilitation, related, among other phases, also to reaching towards the pellet and grasping it. It is possible that we captured the same kind of increased abnormal behavior, since such gestures would translate in our study to failures of reaching, miss or failures of grasping, slip. Further analysis is needed to compare the quality of mistakes pre- and post-lesion. Success rates decreased severely after lesion, as reported widely in literature, and increased over the 2 weeks of rehabilitation, but not to the pre-lesion percentages. Our study focused on intra-rat comparisons, but we also tested for differences between animals. Interestingly, no differences were found between rats based on endpoint features. Thus, endpoint performance features are a useful tool to assess error rates and types of errors on a group level. More importantly, the phase analysis revealed which interval of the reaching and grasping movement was more prone to error. Moreover, this study reveals that different phases of movement are problematic in the timeline of recovery. Miss rates, possibly reflecting the inability to control the forelimb so as to aim at a target, are higher in early stages, whereas slips, caused by inexact grasping, dominate error rates in later stages of recovery. Such insight is essential in developing a rehabilitation strategy that targets specific aspects of movement. With further refinements in movement segmentation, this assessment of behavior outcome could be used to gain insight into neural mechanisms of movement acquisition and execution in healthy subjects, or in mechanisms of recovery, in studies focused on motor impairments.

On the other hand, inspection of trajectories clearly shows independence in strategy between animals, findings supported by other studies that focused on kinematic quantification (Esposito et al., 2014; Guo et al., 2015; Lai et al., 2015). Variability, convex hull and maximum speed increase significantly at the start of rehabilitation with respect to pre-lesion. These differences were not significant with respect to pre-lesion values by the end of rehabilitation. In all features except mean speed, significant differences between rats were detected. Thus, kinematics proved much more sensitive to individual differences than endpoint task outcome, even when they could not capture changes in state between pre- and post-lesion behavior. This result further emphasizes the need for individualized rehabilitation strategies, where kinematics and endpoint behavior measures are used jointly to infer what aspects of movement allow improvement in motor performance. Additional features such as curvature of reaching trajectories might provide further insight into individual strategies of completing the task and into how behavior changes with impairment.

While correlation does not necessarily imply causation, it is still informative to compare the general pattern in kinematics and outcome percentages. We found that variability and convex hull decreased as success rates increased, a relationship significant both in learning and rehabilitation, confirming the idea that kinematics stabilize as endpoint performance increases (Kawai et al., 2015). A high number of attempts, mostly ending in a miss, characterize both the beginning of training and that of rehabilitation and this non-structured pattern of searching for the position of the pellet translates kinematically in high trajectory variability and high convex hull. Thus, it is not surprising that the correlation between convex hull, variability and miss rate, number of attempts, stays significant both in training and rehabilitation. Additionally, more significant relationships of lower magnitude develop during rehabilitation. Interestingly, mean speed was correlated with all endpoint features. Based on observation, animals tended to make fast, imprecise movements in the beginning of rehabilitation, which translated to an indirect correlation of mean speed with success and direct correlation with miss rate and number of attempts. However, the indirect correlations with slip and drop rates may be a by-product of using relative outcome rates. With less misses, the rats proceed further in the task, increasing the probability of making mistakes during further phases, like slips of drops. Thus, we believe further investigations are needed to confirm a true relationship with slip and drop rates. Trajectory length and peak extension decreased with miss and number of attempts in training, an interesting result, suggesting the rats learn to optimize their pellet search with training. This relationship no longer holds in rehabilitation, however, as inverse correlations of trajectory length and peak extension arise with success rate, suggesting optimized pellet search most likely results in overall success. A weak direct correlation of peak limb extension was also found with drop rate, suggesting that longer searches for the pellet, even after a successful grasp, are more likely to result in a drop before the retraction can be successfully completed. Overall, more linear relationships between kinematic parameters and phase outcome arise during rehabilitation, suggesting a change in strategy with respect to training. This is an unsurprising result, since compensation has often been reported as the more prevalent mechanism for achieving improvement after neural damage (Whishaw, 2000; Whishaw et al., 2004; Alaverdashvili and Whishaw, 2008; Alaverdashvili et al., 2008).

Recent review articles assess the important role of compensation in and the lack of features to discriminate it from normal recovery (Kwakkel et al., 2015; Hylin et al., 2017; Jones, 2017). Developing algorithms that can achieve refined detailed behavior description, both robustly and efficiently has become a necessity. Algorithms that assess automatically types of mistakes, not just overall task outcome, together with kinematics of movement may help elucidate the question of what recovery really means and what rehabilitation should focus on: achieving true neural repair and pre-injury behavior quality, or improving ability to adapt to impairments.

## Conclusion

The present study focused on implementing a detailed evaluation of the classical pellet test in a computer program: (i) we developed an algorithm that automatically tracks the movement of the rat’s forelimb using image processing methods; (ii) we expand on existing endpoint behavior features and we assess them along with kinematics of movement, achieving accuracy rates of 86%–92%; (iii) with this extended analysis we captured perturbation of skill after a motor cortical lesion was induced; (iv) analysis of kinematics of movement revealed that rats developed individual strategies to achieve the task; and (v) that learning is distinct from rehabilitation.

## Author Contributions

IN, MD, BN and J-MA designed the research and wrote or revised the manuscript. IN and MD performed experiments and analyzed the data. IN developed the algorithms.

## Conflict of Interest Statement

The authors declare that the research was conducted in the absence of any commercial or financial relationships that could be construed as a potential conflict of interest.
